# Reduced Expression of UPRmt Proteins HSP10, HSP60, HTRA2, OMA1, SPG7, and YME1L Is Associated with Accelerated Heart Failure in Humans

**DOI:** 10.3390/biomedicines13051142

**Published:** 2025-05-08

**Authors:** Petra Bakovic, Vid Mirosevic, Tomo Svagusa, Ana Sepac, Ana Kulic, Davor Milicic, Hrvoje Gasparovic, Igor Rudez, Marjan Urlic, Suncana Sikiric, Sven Seiwerth, Drazen Belina, Matija Bakos, Monika Karija Vlahovic, Rea Taradi, Rado Zic, Ivana Ilic, Borislav Belev, Bosko Skoric, Dora Fabijanovic, Ivo Planinc, Maja Cikes, Filip Sedlic

**Affiliations:** 1 Department of Pathophysiology, School of Medicine, University of Zagreb, 10000 Zagreb, Croatia; petra.bakovic@mef.hr (P.B.); vid.mirosevic@gmail.com (V.M.); borislavbelev@gmail.com (B.B.); 2 Department of Cardiovascular Diseases, Dubrava Clinical Hospital, 10000 Zagreb, Croatia; svagusa.tomo@gmail.com; 3 Department of Pathology, School of Medicine, University of Zagreb, 10000 Zagreb, Croatia; ana.sepac@mef.hr (A.S.); suncana.sikiric@mef.hr (S.S.); sven.seiwerth@mef.hr (S.S.); 4 Ljudevit Jurak Department of Pathology, Sestre Milosrdnice University Hospital Center, 10000 Zagreb, Croatia; 5 Department of Oncology, University Hospital Centre Zagreb, 10000 Zagreb, Croatia; ana.kulic100@yahoo.com; 6 School of Medicine, University of Zagreb, 10000 Zagreb, Croatia; davor.milicic@kbc-zagreb.hr (D.M.); bskoric3@yahoo.com (B.S.); maja.cikes@gmail.com (M.C.); 7 Department of Cardiovascular Diseases, University Hospital Centre Zagreb, 10000 Zagreb, Croatia; dora.fabijanovic@gmail.com (D.F.); ivo.planinc@gmail.com (I.P.); 8 Department of Surgery, School of Medicine, University of Zagreb, 10000 Zagreb, Croatia; hgasparovic@gmail.com (H.G.); rudi@kbd.hr (I.R.); davorz123@yahoo.com (R.Z.); 9 Department of Cardiac Surgery, University Hospital Center Zagreb, 10000 Zagreb, Croatia; urlic.marjan@gmail.com (M.U.); drazen.belina@hotmail.com (D.B.); 10 Department of Cardiac and Transplant Surgery, Dubrava Clinical Hospital, 10000 Zagreb, Croatia; 11 Department of Pathology and Cytology, University Hospital Centre Zagreb, 10000 Zagreb, Croatia; rea.taradi@gmail.com (R.T.); ricilic@gmail.com (I.I.); 12 Department of Pediatrics, University Hospital Centre Zagreb, 10000 Zagreb, Croatia; mbakos11@gmail.com; 13 DNA Laboratory, Department of Forensic Medicine and Criminology, School of Medicine, University of Zagreb, 10000 Zagreb, Croatia; monika.karija.vlahovic@mef.hr; 14 Department of Plastic, Reconstructive and Aesthetic Surgery, Dubrava University Hospital, 10000 Zagreb, Croatia

**Keywords:** heart, mitochondria, UPRmt, ROS, cardiomyopathy

## Abstract

**Background/Objectives:** The mitochondrial unfolded protein response (UPRmt) is one of the mitochondrial quality control mechanisms that is responsible for reparation and removal of damaged proteins in mitochondria. **Methods**: Here we investigated the role of the UPRmt in the myocardium of humans with and without heart failure and in the cell culture model. **Results**: The analysis of myocardial samples by ELISA from patients with ischemic cardiomyopathy (ICM) and dilated cardiomyopathy (DCM), as well as healthy donors, revealed a significantly reduced expression of the UPRmt proteins HSP10, CLPP, LONP1, OMA1, and SPG7 in patients with DCM and ICM. Furthermore, patients with DCM and ICM exhibited elevated levels of myocardial reactive oxygen species (ROS, tested by 4-hydroxynonenal) compared to controls, and a positive correlation between ROS production and mt-HSP70, OMA1, and SPG7 protein expression. The correlation analysis indicated a negative correlation between cardiomyocyte hypertrophy and the expression of several UPRmt genes. The inhibition of four tested UPRmt effector proteins exacerbated the injury of cultured cells under oxidative stress. The patients with ICM, DCM, or both, who showed lower myocardial expression of HSP10, HSP60, HTRA2, OMA1, SPG7, and YME1L, underwent heart transplantation or implantation of a left ventricular assist device earlier in life compared to those with the higher protein expression. **Conclusions**: In conclusion, our findings indicate that the reduced expression of several UPRmt effector proteins is associated with accelerated heart failure in patients, which, together with other results, indicates that impaired UPRmt may contribute to the pathogenesis of heart failure in humans.

## 1. Introduction

Heart failure (HF) is a heterogeneous clinical syndrome that remains a major cause of mortality and morbidity, and it is most commonly caused by ischemic cardiomyopathy (ICM) or dilated cardiomyopathy (DCM) [1,2]. Obstructive coronary artery disease is a dominant cause of ICM [1]. On the other hand, DCM has a multifactorial etiology that is non-ischemic in origin with a predominantly genetic basis and is characterized by an increase in the size of the left ventricle and systolic dysfunction, unexplained by abnormal loading conditions [2,3]. One of the key characteristics of patients undergoing heart transplantation or left ventricular assist device (LVAD) implantation is reduced ejection fraction. Mitochondrial dysfunction and excessive production of reactive oxygen species (ROS) are key underlying factors in the pathogenesis of HF [4].

The mitochondrial unfolded protein response (UPRmt) is a homeostatic mechanism aimed at maintaining the mitochondrial proteome and thereby proper mitochondrial function [5]. It is usually activated after disturbed mitochondrial proteostasis and acts to refold misfolded proteins by chaperones such as HSP10, HSP60, or mt-HSP70, or to remove them by proteases like YME1L, OMA1, LONP1, CLPP, SPG7, and HTRA2 [6]. Our recent study demonstrated a reduced mitochondrial biomass in failed human hearts [7]. Moreover, the expression of a large number of genes belonging to the mitochondrial quality control (MQC) was also reduced in the myocardium of patients with HF, suggesting an impaired mitochondrial function [7]. This included effector and regulatory genes of the UPRmt as one of the MQC branches. However, it is not clear whether the reduced UPRmt gene expression translates into the downregulation of UPRmt proteins since protein turnover, such as protein degradation by proteasomes, is altered in failing hearts [8].

It is well established that impaired UPRmt can lead to HF. Different in vivo and in vitro stresses in the mammalian myocardium proved to activate UPRmt, which acted to ameliorate mitochondrial dysfunction and improve cardiomyocyte viability under stress conditions found in HF [9]. For example, Yme1l knockout mice develop HF early in life [10]. Moreover, the overexpression of LONP1 or other UPRmt effector proteins protects the heart from ischemia–reperfusion [11]. However, there are also studies that show deleterious effects of UPRmt in the heart [12,13,14].

Thus, we have designed this study to investigate the association between the myocardial expression of several effector UPRmt proteins and the age when patients underwent heart transplantation or LVAD implantation (age_HTx/LVAD_) and to test the effect of UPRmt elements on the cell viability, myocardial ROS generation, and cardiomyocyte hypertrophy.

## 2. Materials and Methods

### 2.1. Human Myocardial Samples

The demographic and clinical parameters of patients with HF and control subjects are shown in Table 1 and Table 2. All samples were obtained from the apex of the left ventricle. The middle part of the transmural section was used for all analyses. The myocardial samples from patients with advanced stage of HF, including 30 patients with DCM and 29 patients with ICM, were acquired immediately after heart transplantation (44 samples) or LVAD implantation (15 samples) at the University Hospital Centre Zagreb and the Dubrava University Hospital (Zagreb, Croatia). All patients had HF with reduced ejection fraction (Table 2). These 59 samples were used for the 4-hydroxynonenal (4HNE) and UPRmt protein expression analyses. Following heart explantation or apical coring for LVAD implantation, left ventricular myocardium samples were excised and divided in half. One part was placed in the formalin for later pathohistological analyses, and the other part was transported in a cold, oxygenated cardioplegic solution and then stored at −80 °C until further analysis. Six control left ventricle myocardial samples were obtained from human donors without known cardiac disease by the National Disease Research Interchange (Philadelphia, PA, USA). They had no signs of heart disease and no medical history of heart disease. The study’s exclusion criteria were the following: primarily structural heart abnormalities, diabetes mellitus, and patients under 40 years old to exclude cardiomyopathies that were driven genetically with an early onset and high expressivity [15,16]. A left ventricular ejection fraction below 35% coupled with significant coronary artery disease confirmed via angiography were criteria for ICM. For DCM, the criteria included a left ventricular ejection fraction below 35% alongside an increase in left ventricular dimensions in the absence of coronary artery disease or abnormal loading conditions. Each participant gave written informed consent to participate in the study. All procedures were conducted in accordance with the Declaration of Helsinki. The ethical approval for the study was obtained from the ethical committees of the University of Zagreb School of Medicine (approval number: 380-59-10106-18-111/55; 22 March 2018), University Hospital Center Zagreb (approval number: 02/21 AG), and the Dubrava University Hospital (approval number: 2017/2310-05).

### 2.2. Protein Quantification by ELISA

We selected ELISA (Thermo Fisher Scientific, Waltham, MA, USA) for protein quantification over Western blot and immunohistochemistry due to the need for high throughput and high reliability [17,18]. Approximately 500 mg of tissue from each sample was ground into a fine powder using liquid nitrogen and a pestle and mortar. Tissue protein extraction reagent (T-PER, Thermo Scientific, Waltham, MA, USA) with protease inhibitor (Halt Protease Inhibitor Cocktail, Thermo Scientific, Waltham, MA, USA) was added to the grounded tissue in a ratio of 1:5, w:v. Afterwards, homogenate was vortexed, sonicated in an ultrasonic bath (3 × 10 s), and centrifuged (4000 rpm/+4 °C/15 min). Supernatants were aliquoted and stored at −80 °C until further analysis. Total protein levels were determined by the Bradford method. Commercially available enzyme-linked immunosorbent assays (ELISAs) were used to determine the levels of HTRA2 (#EHHTRA2) and HSP60 (#EH244RB), both from Invitrogen, Thermo Scientific, Waltham, MA, USA; and HSP10 (#abx573810), LONP1 (#abx388296), OMA1 (#abx389971), SPG7 (#abx546984), mt-HSP70 (#abx350060), CLPP (#abx251576), YME1L (#abx550672), TOMM20 (#abx384347), and 4HNE (#abx257639), all from Abbexa, Cambridge, UK. The assay sensitivities were 200 pg/mL, 0.41 ng/mL, 75 pg/mL, <18.8 pg/mL, <9.38 pg/mL, <0.135 pg/mL, 0.47 ng/mL, <0.54 ng/mL,0.11 ng/mL, <0.14 ng/mL, and 0.38 ng/mL, respectively.

### 2.3. Cell Culture

Human aortic endothelial cells (HAEC, CC-2535, Lonza, Basel, Switzerland) were cultured according to the manufacturer’s recommendation (Lonza, Basel, Switzerland) in EBM-2 medium with the addition of appropriate supplements in an incubator at a temperature of 37 °C with a humidified atmosphere containing 5% CO_2_. Before the experiments, cells were cultured in 25 cm^2^ and 75 cm^2^ bottles. For each experiment, the adherent culture of confluent cells was detached from the surface using a 0.25% trypsin-EDTA solution (Lonza, Switzerland) for 3 min, and cells were counted using the trypan blue staining method on a Neubauer-type hemocytometer. The cells were seeded into 96-well plates. Antimycin A (12.5 µM; #A8674, Sigma-Aldrich, St. Louis, MI, USA) was used to induce oxidative stress. We also investigated the effects of pharmacological inhibitors of specific UPRmt proteins: 2-cyano-3,12-dioxo-oleana-1,9(11)-dien-28-oic acid methyl ester (CDDO, 1 µM; #SML3452), epigallocatechin-3-gallate (EGCG, 30 µM, #E4143), nonactin (10 µM, #N2286), and MKT-077 (0.8 µM, #M5449 all from Sigma-Aldrich, St. Louis, MI, USA, SAD). One day after seeding, the cells were exposed to treatment with antimycin A alone or together with one of the UPRmt inhibitors for three days. On the fourth day, the viability of the cells was analyzed using the 3-(4,5-dimethylthiazol-2-yl)-2,5-diphenyltetrazolium bromide (MTT) assay (EZ4U, Biomedica) that was added to the treated cells. Viable cells with active metabolism convert MTT into formazan, generating a signal that was read on a spectrophotometer (Awareness Technology Stat Fax 3200 Microplate Reader). The absorbance was measured at 590 nm, reporting the number of viable cells. The concentrations of tested inhibitors were selected according to previous reports [19,20,21,22].

### 2.4. Pathological Analysis

The formalin-fixed and paraffin-embedded samples of myocardium were stained routinely by hematoxylin and eosin and examined by light microscopy. In longitudinally sectioned cardiomyocytes, the diameter was measured at the widest part of the cell and analyzed for 5–10 cells per slice. The average diameter was calculated for each patient as a marker of hypertrophy. For these analyses, all samples were acquired from the middle part of the section obtained from the apex. The average coefficient of variation for the measured diameter in one sample was 0.098.

### 2.5. Quantitative PCR

For the analyses of correlation between the gene expression and cell hypertrophy, we used the gene expression data from 20 patients with either DCM or ICM that underwent heart transplantation or LVAD implantation and that were previously published by us [7]. Please see [7] for details.

### 2.6. Statistical Analysis

Statistical analyses were performed using GraphPad Prism v8.4.3 (GraphPad Software, San Diego, CA, USA). The normality of data distribution was tested with a D’Agostino–Pearson omnibus test and the presence of outliers with the Grubbs test. Bivariate correlations were analyzed with the Pearson correlation coefficient in the case of normally distributed data or the Spearman correlation coefficient in the case of non-normally distributed data. For the comparison of three groups, normally distributed data were analyzed using one-way analysis of variance (1w-ANOVA) followed by a Tukey post hoc test. The non-normally distributed data were analyzed using the Kruskal–Wallis (KW) test followed by Dunn’s post hoc test. The means between two groups were compared with the two-tailed unpaired Student’s *t*-test or Mann–Whitney U-test, as appropriate. A multilinear regression analysis was conducted, incorporating each protein that exhibited a significant correlation with 4HNE (HSPA9, OMA1, SPG7, and TOMM20) as dependent variables. The independent variables included 4HNE, as well as patients’ smoking habits and sex, with the analysis aimed at identifying potential outliers.

## 3. Results

### 3.1. UPRmt Proteases and Chaperones Are Downregulated in Failing Human Myocardium

**Figure 1:**
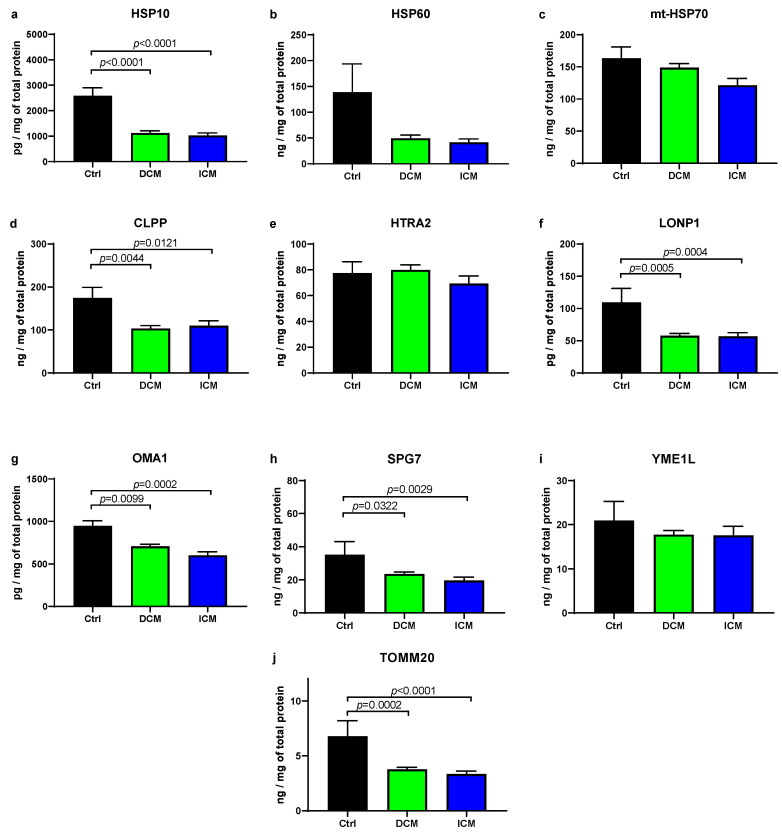
Protein expression of UPRmt elements and TOMM20 in failing human hearts. (**a**–**j**) Protein levels in human myocardial samples were quantified using ELISA kits. Analyses included UPRmt chaperones (HSP10, HSP60, and mt-HSP70), proteases (CLPP, HTRA2, LONP1, OMA1, SPG7, and YME1L), and translocase of the outer mitochondrial membrane TOMM20, which was used to assess mitochondrial content in the myocardium. Summary data are presented as mean value ± SEM. Abbreviations: Ctrl (control; n = 6); DCM (heart failure caused by dilated cardiomyopathy; n = 30); and ICM (heart failure caused by ischemic cardiomyopathy; n = 29). Significant *p*-values are indicated in graphs.

We analyzed the levels of heat shock proteins involved in the UPRmt, which are located in the mitochondrial matrix (Figure 1). Compared to the control, the level of HSP10 was significantly decreased in both DCM (DCM/Ctrl = 0.28) and ICM (ICM/Ctrl = 0.23) groups. Conversely, the level of mt-HSP70 was unaltered in both groups. Additionally, HSP60 showed only a tendency to decrease in the DCM and ICM groups. It cannot be excluded with certainty that with more than six control samples, this difference would be significant. Four out of six analyzed proteases of UPRmt displayed a significant decrease in protein level in DCM and ICM samples compared to control, including CLPP (DCM/Ctrl = 0.59; ICM/Ctrl = 0.63), SPG7 (DCM/Ctrl = 0.66; ICM/Ctrl = 0.55), LONP1 (DCM/Ctrl = 0.54; ICM/Ctrl = 0.51), and OMA1 (DCM/Ctrl = 0.74; ICM/Ctrl = 0.63). However, HTRA2 and YME1L showed no difference in either group. Additionally, we analyzed the protein level of TOMM20, a translocase of the inner mitochondrial membrane, and an indicator of mitochondrial biomass [7]. It was significantly decreased in the DCM (DCM/Ctrl = 0.55) and ICM (ICM/Ctrl = 0.45) groups compared to control.

Overall, these results indicate reduced protein levels of several UPRmt chaperones and proteases in failing hearts, as well as reduced mitochondrial biomass.

### 3.2. Human Failing Hearts Produce More ROS, Which Positively Correlate with Several UPRmt Proteins

**Figure 2:**
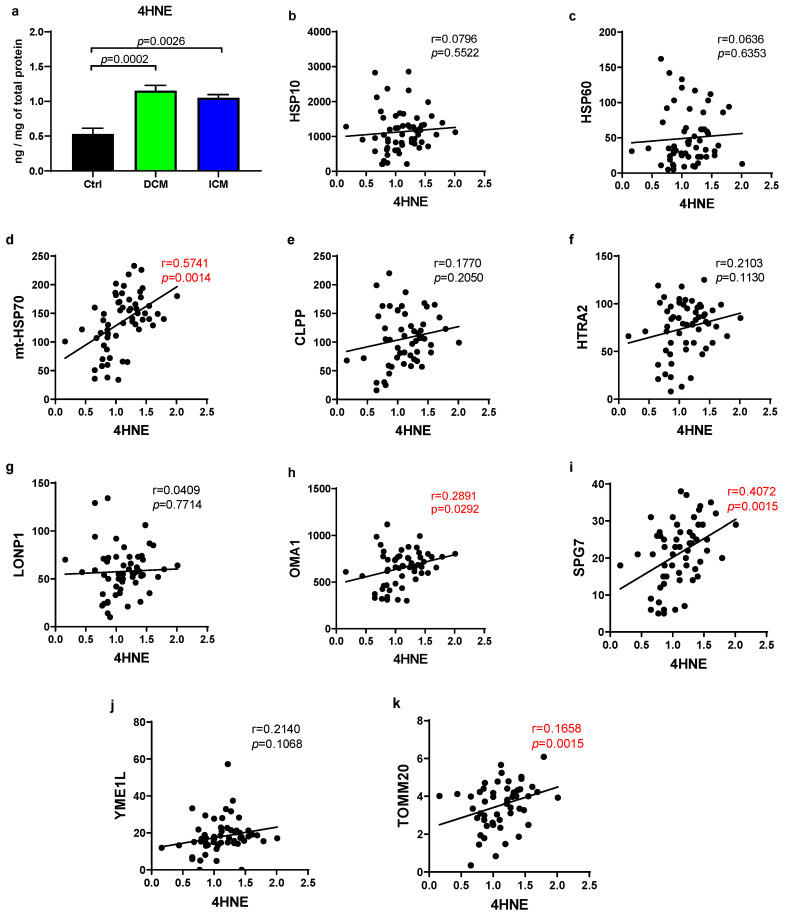
Production of ROS and UPRmt in human hearts. (**a**) Levels of 4-hydroxynonenal (4HNE) were quantified using an ELISA kit in samples of failing human hearts. (**b**–**k**) Scatter plots illustrating a correlation between 4HNE levels and UPRmt protein levels in failing human hearts. Pearson or Spearman coefficient correlation and *p*-value are given on each graph, where red color indicates a statistically significant correlation (*p* < 0.05). Abbreviations: Ctrl (control; n = 6); DCM (heart failure caused by dilated cardiomyopathy; n = 30); and ICM (heart failure caused by ischemic cardiomyopathy; n = 29).

A highly toxic and most abundant byproduct of lipid peroxidation, 4HNE exhibits high reactivity and the capacity to covalently bind to proteins, thereby rendering them inactive and contributing to cytotoxicity [23,24]. Since it is a good marker of ROS generation, we quantified it to evaluate oxidative stress in cardiac tissue. As shown in Figure 2, there was a notable elevation in 4HNE levels observed in both DCM (DCM/Ctrl = 2.18) and ICM (ICM/Ctrl = 1.98) groups when compared to the control, highlighting the presence of heightened oxidative stress in failing human hearts. However, there was no significant difference between the DCM and ICM groups.

We analyzed the relationship between the 4HNE levels and various UPRmt proteins, alongside TOMM20 in cardiac tissue from all HF patients combined. Protein TOMM20 demonstrated a significant positive correlation with 4HNE (*r* = 0.1658, *p* = 0.0015), as did three out of nine UPRmt elements tested. Notably, among these, one chaperone mt-HSP70 (*r* = 0.5741, *p* = 0.0014) displayed a particularly strong positive correlation, distinguishing it from the proteases SPG8 (*r* = 0.4072, *p* = 0.0015) and OMA1 (*r* = 0.2891, *p* = 0.0292). No correlation was observed for other UPRmt elements. These findings suggest that ROS production is elevated in the myocardium of failing hearts and that oxidative stress is associated with upregulation of several UPRmt elements [25]. A multilinear regression analysis was performed (Table 3), using proteins significantly correlated with 4HNE (HSPA9, OMA1, SPG7, and TOMM20) as dependent variables. Independent variables included 4HNE levels, smoking status, and patient’s sex, with the aim of assessing their associations with protein levels. Concentration of 4HNE was significantly associated with HSPA9, SPG7, and TOMM20. Smoking showed no significant relationships, while sex was a significant predictor only for SPG7.

### 3.3. UPRmt Genes Display Negative Correlation with the Degree of Cardiomyocyte Hypertrophy

**Figure 3:**
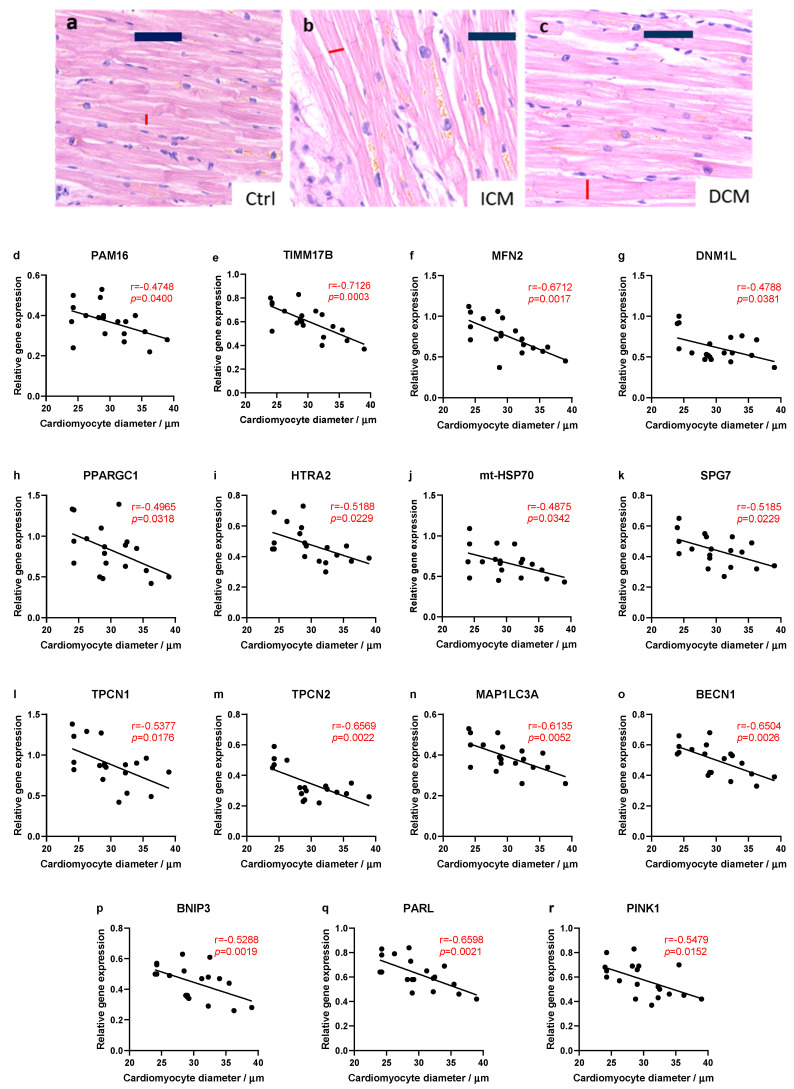
Relationship between myocardial hypertrophy and the UPRmt and MQC gene expression. (**a**–**c**) Representative images of hematoxylin and eosin (H&E)-stained sections of myocardium that were used to measure cardiomyocyte diameters in formalin-fixed paraffin-embedded slices of hearts of patients with DCM or ICM. Scale bars = 50 µm (**d**–**r**) Scatter plots illustrate the correlation between cardiomyocyte’s diameter and a relative gene expression in failing human hearts (ICM and DCM samples combined). Pearson or Spearman coefficient correlation and *p*-values are given on each graph, where red color indicates statistically significant difference (*p* < 0.05). Abbreviations: Ctrl (control; n = 6); DCM (heart failure caused by dilated cardiomyopathy; n = 30); and ICM (heart failure caused by ischemic cardiomyopathy; n = 29).

In some samples used in this study, we previously analyzed the expression of UPRmt and other MQC genes by quantitative real-time PCR [7]. Here we used those data to analyze the correlation between the UPRmt and MQC gene expression and the extent of cardiomyocyte hypertrophy in failing hearts (Figure 3). It has been previously shown in many studies that the extent of cardiomyocyte hypertrophy, acting as a compensatory mechanism, is in correlation with the severity of heart insufficiency [26]. Out of 49 tested genes, *PAM16*, *TIMM17B*, *MFN2*, *DNM1L*, *PPARGC1*, *HTRA2*, *HSPA9*, *SPG7*, *TPCN1*, *TPCN2*, *MAP1LC3A*, *BECN1*, *PINK1*, *BNIP3,* and *PARL* genes were in statistically significant and negative correlation with the cardiomyocyte diameter, i.e., the extent of cardiac hypertrophy. Genes *HTRA2* and *HSPA9* (mt-HSP70 protein) belong to the UPRmt. Interestingly, we observed only negative correlations. This indicates that the reduction in the expression of several UPRmt genes is associated with cardiac hypertrophy, i.e., severity of heart insufficiency. There were no significant differences in the expression of MQC genes between the DCM and ICM samples [7].

### 3.4. Inhibition of UPRmt Proteases and Chaperones Reduces Survival of Stressed Cells

To test whether the activity of UPRmt chaperones and proteases is detrimental or beneficial in stress conditions, we exposed the cultured cells to oxidative stress with or without UPRmt inhibitors. To induce oxidative stress, we used antimycin A, which promotes the leakage of electrons from the complex III of the electron transport chain, inducing production of ROS.

**Figure 4:**
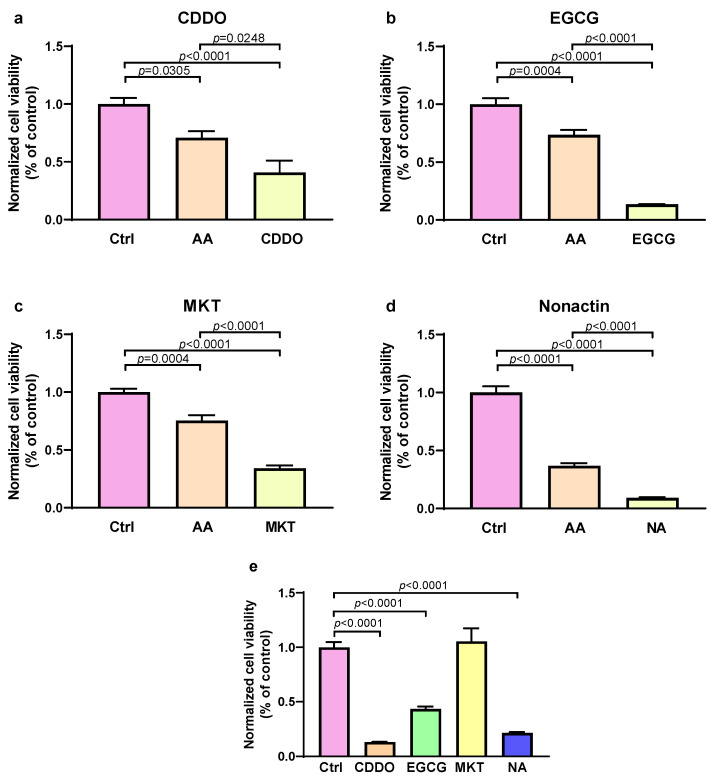
Effect of UPRmt inhibitors on human cultured cells exposed to oxidative stress. An MTT assay was used to assess viability of cells without any treatment (control, Ctrl), cells treated with antimycin A to induce oxidative stress (AA), and cells treated with combination of AA and one of four different UPRmt inhibitors: (**a**) EGCG (inhibitor of OMA1); (**b**) MKT-077 (MKT; inhibitor of mt-HSP70); (**c**) CDDO (inhibitor of LONP); and (**d**) nonactin (NA; inhibitor of HSP60). Effects of inhibitors alone, without AA are shown in (**e**). Data are expressed as means ± SEM with n = 8 for each group. Data are normalized to control (Ctrl). Significant *p*-values are indicated in graphs.

As shown in Figure 4a–d, treatment with antimycin A in combination with either EGCG (OMA1 inhibitor), CDDO (LONP1 inhibitor), nonactin (HSP60 inhibitor), or MKT-077 (mt-HSP70 inhibitor) reduced the survival of cultured cells by 40%, 70%, 74%, and 41%, respectively, compared to cells exposed only to antimycin A. This indicates that the inhibition of all tested UPRmt effector proteins exacerbated cell injury, suggesting that UPRmt has a protective effect in stressed cells. Application of inhibitors alone, without stress showed either no effect (MKT-077), or reduced cell viability, but not as much as inhibitors with stress (EGCG) or substantial reduction of cell viability (CDDO and nonactin), as shown in Figure 4e.

### 3.5. Patients with Lower Expression of UPRmt Proteins Undergo Heart Transplantation or LVAD Implementation at Younger Age

**Figure 5:**
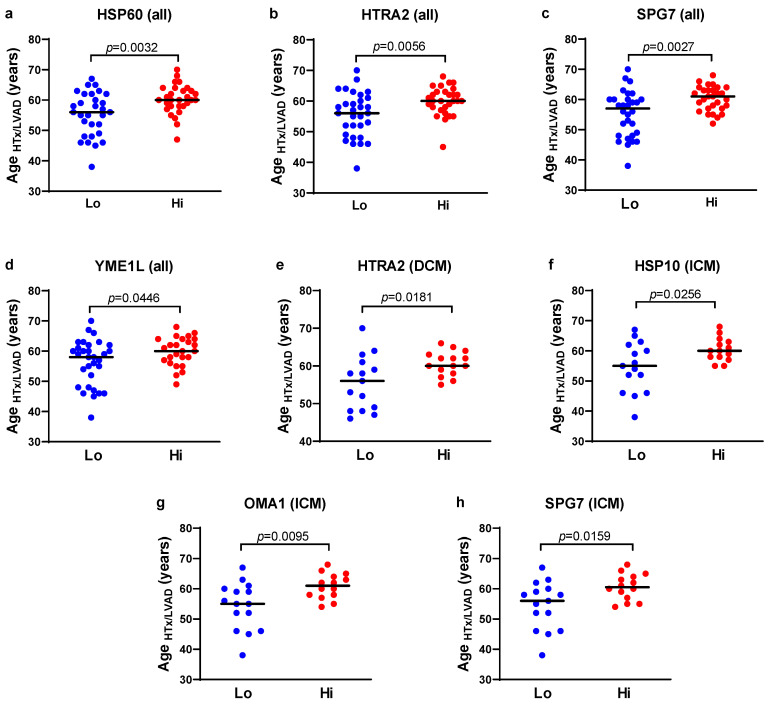
The age of patients undergoing heart transplantation or LVAD implantation according to the myocardial expression of UPRmt proteins. (**a**–**h**) Individual data points represent the age when patients underwent heart transplantation or LVAD implantation (age_HTx/LVAD_), and the horizontal line indicates mean values. Patients were divided into two groups according to the myocardial expression of respective UPRmt proteins (CLPP, HSP10, HSP60, mt-HSP70, HTRA2, LONP1, OMA1, SPG7, TOMM20, and YME1L), where half of the patients with a lower expression were assigned to the low group (Lo) group, and half of the patients with a higher expression were assigned to the higher group (Hi). The patients with dilated cardiomyopathy (DCM) and ischemic cardiomyopathy (ICM) were analyzed separately or together (all). Only significant differences (*p* < 0.05) are shown.

Next, we investigated the association between the myocardial UPRmt protein expression and the age when patients received heart transplantation or LVAD implantation (age_HTx/LVAD_) as an indication of HF progression (Table 4 and Figure 5). According to the myocardial concentration of each protein, one half of the patients with a lower protein expression were assigned to the lower group (Lo), and the other half with a higher protein expression were assigned to the higher group (Hi). When analyzing all patients with heart failure (ICM and DCM combined), a significantly lower age_HTx/LVAD_ was observed in those who had lower HSP60 (average age of transplantation reduced by 4.9 years), HTRA2 (average age_HTx/LVAD_ reduced by 4.6 years), SPG7 (average age_HTx/LVAD_ by 5.0 years), and YME1L (average age_HTx/LVAD_ reduced by 3.6 years). Trends for earlier need for heart transplantation or LVAD implantation were also observed for lower CLPP and OMA1 expressions. Patients with DCM and lower myocardial HTRA2 expression had lower age_HTx/LVAD_ by 5.1 years, and lower SPG7 displayed only a trend for reduced age_HTx/LVAD_. Patients with ICM who had reduced myocardial HSP10, OMA1, and SPG7 had age_HTx/LVAD_ reduced by 5.8, 6.6, and 6.2 years, respectively, and lower HSP60 and YME1L displayed only trends for reduced age_HTx/LVAD_. Altogether, six out of nine tested UPRmt effector proteins exhibited significantly lower expression in patients who underwent heart transplantation or LVAD implantation earlier in life, either in ICM, DCM, or HF combined groups. Neither protein was more expressed in the earlier heart transplantation/LVAD implantation group. TIMM20 and ROS generation showed no differences in the age_HTx/LVAD_ between the lower and higher expressing groups. This is in agreement with the data presented in Figure 4 showing that the inhibition of UPRmt proteins reduced the survival of stressed cells.

## 4. Discussion

Here we showed that the failing myocardium of patients with ICM and DCM displayed downregulation of UPRmt effector proteases CLPP, LONP1, OMA1, SPG7, and effector chaperone HSP10, compared to control myocardium. The generation of ROS was elevated in the ICM and DCM myocardium compared to control hearts. The expression of UPRmt proteases SPG7, OMA1, and chaperone mt-HSP70 was in positive correlation with ROS generation in the failing myocardium. The expression of 15 MQC genes, which included two UPRmt genes, *HSPA9* and *HTRA2*, was in negative correlation with the diameter (hypertrophy) of cardiomyocytes in the failing human myocardium. Inhibition of UPRmt effector proteins, OMA1, LONP1, HSP60, and mt-HSP70, potentiated human cell death induced by oxidative stress. Patients with HF having lower myocardial expression of HTRA2, SPG7, YME1L, and HSP60 underwent heart transplantation or LVAD implantation at a younger age compared to those with a higher expression of respective proteins. Patients with ICM and lower myocardial expression of HSP10, OMA1, and SPG7, and patients with DCM and lower myocardial expression of HTRA2, also underwent heart transplantation at a younger age compared to those with a higher expression of respective proteins.

Previously, we showed that many MQC genes, which included UPRmt genes, *DDIT3, UBL5, EIF2AK4, HSPA9* (mt-HSP70 protein)*, HSPE1* (HSP10 protein)*, YME1L, LONP1, SPG7, HTRA2,* and *OMA1*, were downregulated in the failing myocardium of patients with either ICM or DCM [7]. Since gene expression may not fully represent protein expression in the failing heart due to impaired protein degradation [8], and since protein expression is more relevant for the phenotype of the organ, here we examined the expression of selected UPRmt proteins. We found that CLPP, HSP10, LONP1, OMA1, and SPG7 proteins were downregulated in HF myocardium, and the same genes were also downregulated in our previous study (*CLPP* was not tested) [7]. However, we also noticed some discrepancies between gene and protein expression, which may stem from the above-mentioned changes in protein degradation in the failing heart or from the use of different myocardial samples in these two studies. Here we observed that HSP60 protein showed a trend to be downregulated in HF, whereas its gene was not altered in the previous study, while *HSPA9* gene was downregulated, but its protein mt-HSP70 was not altered in this study. Due to a relatively low number of controls (six), we cannot eliminate the possibility of actual significance of this difference observed for the HSP60 protein. Overall, the majority of UPRmt genes and proteins tested in these two studies followed the same pattern of change, i.e., downregulation in DCM- and ICM-induced HF. Since previous studies showed that UPRmt has a protective role against various forms of stress [27,28], including its cardioprotective properties [6], the data presented here suggest that downregulation of UPRmt effector elements may contribute to HF in these patients. The clinical significance of this finding was further addressed by analyzing the age_HTx/LVAD_, as discussed later. No significant differences were observed between DCM and ICM myocardial samples. This is in accordance with our previous study analyzing MQC gene expression in failing heart samples, where almost all tested (>40) genes were similarly expressed between ICM and DCM.

Here we showed that myocardial ROS generation was elevated in DCM and ICM compared to control. This is in agreement with studies showing elevated ROS generation in HF [29,30,31]. We have shown before that excessive ROS generation induces the death of cardiomyocytes [32], which involves mitochondrial calcium overload [33] and the opening of the mitochondrial permeability transition pore [34,35]. Studies published by others also show that mitochondrial ROS contributes to angiotensin II-induced cardiac hypertrophy and HF [36], promotes myocardial ischemia/reperfusion injury [37], and induces cardiomyocyte pyroptosis in DCM [38]. However, ROS can elicit protective mechanisms in cardiomyocytes, especially against ischemic injury [39,40]. Interestingly, ROS generation was in positive correlation with the expression of three UPRmt proteins (mt-HSP70, OMA1, and SPG7), which indicates an association between ROS production and the expression of these UPRmt elements. These results were confirmed using the multilinear analysis where only 4HNE was a significant predictor of protein expression in three out of four tested proteins. Only SPG7 showed significant associations with both 4HNE and sex, but the absence of multicollinearity suggests that sex is not a confounder, but an independent predictor of SPG7 levels. It has been shown before that oxidative stress can upregulate UPRmt [41,42], which would suggest that ROS induced UPRmt in failing human hearts in our study.

Mitochondria play crucial roles in the pathological processes of myocardial injury and therefore they serve as a potential target for myocardial protection. Consequently, ensuring the stability and proper folding of mitochondrial proteins is of key importance. During ischemia–reperfusion stress, mitochondrial proteins may misfold, triggering the activation of the UPRmt [43]. This response facilitates the folding of proteins within the mitochondrial proteome, contributing to the MQC [43]. Analysis of the correlation between the MQC gene expression, which we have published recently [7], and the extent of cardiomyocyte hypertrophy showed a relatively consistent trend. A greater degree of cardiac hypertrophy correlated negatively with the expression of MQC and UPRmt genes, including *PAM16*, *TIMM17B*, *MFN2*, *DNM1L*, *PPARGC1*, *HTRA2*, *HSPA9*, *SPG7*, *TPCN1*, *TPCN2*, *MAP1LC3A*, *BECN1*, *BNIP3*, *PARL,* and *PINK1*. It is well established that cardiac hypertrophy is a compensatory response to insufficient function of cardiomyocytes and that it is associated with the degree of cardiac insufficiency [44,45]. Thus, our data suggest that a reduced expression of these UPRmt proteins may result in a greater cardiac dysfunction and subsequent hypertrophy. All of our patients had NYHA III or IV status, and gene expression showed no difference relative to the NYHA status, potentially due to issues with the reproducibility of NYHA classification [46]. We corroborated our findings with cell culture experiments, where we showed that the inhibition of all four tested effector UPRmt elements (HSP60, mt-HSP70, LONP1, and OMA1) augmented cell injury induced by oxidative stress. This indicates that UPRmt has a protective effect in oxidative stress.

Inhibition of the mt-HSP70 did not affect the viability of unstressed cells, suggesting its importance only during stress conditions. Inhibition of the OMA1 reduced the viability of unstressed cells and even more of stressed cells, indicating its importance in normal and even more in stressed conditions. Moreover, the inhibition of HSP60 and LONP1 reduced the viability of unstressed and stressed cells to a similar extent, suggesting similar importance of these UPRmt proteins in normal and stress conditions.

The protective role of the UPRmt observed here is in line with studies published by others. For example, Shi et al. [47] recently published the results of research in which they used a transverse aortic constriction mouse model of pressure overload-induced hypertrophy and heart failure. They showed that *CTRP3* overexpression induced UPRmt activation during pathologic cardiac hypertrophy and alleviated mitochondrial dysfunction and oxidative stress. Furthermore, Smyrnias et al. [9] demonstrated that pharmacological enhancement of UPRmt, which is activated during chronic pressure overload, alleviated both mitochondrial and contractile dysfunction in the stressed heart. The induction of UPRmt can also protect from ischemia–reperfusion injury [48]. In the study by Smyrnias et al. [9], the authors found elevated expression of UPRmt genes in patients with severe aortic stenosis. It is possible that they observed upregulation of the UPRmt as a compensatory response in hearts that were not in the terminal stage of the disease requiring heart transplantation or LVAD implantation. We showed downregulation of UPRmt proteins likely because our patients were in the terminal stage of heart diseases where compensatory UPRmt activation could not be elicited.

Importantly, we found that six out of nine tested UPRmt proteins were downregulated in the myocardium of patients (with either ICM, DCM, or all HF patients) who underwent heart transplantation or LVAD implantation earlier in life. These included HSP10, HSP60, HTRA2, OMA1, SPG7, and YME1L. One more (CLPP) also showed a trend for reduced expression with accelerated HF. It indicates the cardioprotective effects of UPRmt and corroborates our other findings presented here. However, some studies demonstrated that UPRmt elements are associated with heart damage, including eIF2α and cardiomyocyte apoptosis and heart failure [49], OMI and cardiomyocyte apoptosis [13], SPG7 and coronary artery disease [50], and HSP60 and heart failure [51]. It is possible that moderate UPRmt activation is cardioprotective, acting to repair or remove misfolded proteins, while its excessive action becomes deleterious to the heart, causing unbalanced protein destruction and acting in a non-linear fashion [52].

Our study has several limitations. Part of the study are cross-sectional analyses that do not allow firm conclusions about the causality between tested variables that showed significant associations. However, cell culture experiments supported our findings by demonstrating the causality between UPRmt inhibition and enhanced cell death. We may not have observed some associations with moderate effects due to a limited number of human control myocardial samples (six) that are hard to obtain. Thus, due to a relatively limited number of control samples, the results should be interpreted with caution pertaining to generalizability and the possibility of making type II errors. Since this is not an interventional study, we did not analyze the duration between the first diagnosis of coronary artery disease and the heart transplantation or LVAD implantation for the assessment of HF progression. Instead, we analyzed the age_Htx/LVAD_, which allowed us to pool together patients with ICM and DCM. Moreover, for patients with ICM, the first diagnosis of coronary artery disease is usually set after the major cardiac incident, which does not truly represent the onset of ischemic heart disease. One of the limitations is the use of HAECs that are not cardiomyocyte models. We used them because they are human and completely mature cells. The mitochondria of endothelial cells and cardiomyocytes share many similarities [53]. We did not use cardiomyocyte models due to the need to culture cells for several days, excluding primary cardiomyocytes that undergo phenotypic changes with culturing [54]. We also wanted to use human cells to eliminate species differences in mitochondrial function [55] and to use adult cell types to avoid issues arising from cell immaturity and heterogeneity in differentiated cardiomyocytes [56,57].

In conclusion, we found that the UPRmt is downregulated in the myocardium of patients with HF and that patients with a reduced expression of UPRmt elements undergo heart transplantation or LVAD implantation earlier in life. With observations that UPRmt inhibition increased cell damage in cell culture models and that the extent of myocardial hypertrophy is associated with the downregulation of UPRmt gene expression, these findings strongly suggest that UPRmt is an important cardioprotective mechanism and that its downregulation may be in part responsible for accelerated HF in patients. Moreover, increased myocardial ROS generation is associated with an increased expression of UPRmt proteins.

## Figures and Tables

**Table 1:** Clinical parameters of participants in control and HF groups obtained prior to sampling of myocardium.

	Non-HF	HF	*p*-Value
n	6	59	
Age (y), mean ± SEM	56 ± 2	57 ± 1	NS
Female gender	2 (33%)	8 (13%)	NS
Height (cm), mean ± SEM	179 ± 5	176 ± 9	NS
Body mass (kg), mean ± SEM	104 ± 11	82 ± 14	NS
BMI (kg/m^2^), mean ± SEM	32.9 ± 4.5	26.3 ± 3.6	NS
BSA (m^2^), mean ± SEM	2.3 ± 0.1	2.0 ± 0.2	NS
Smoking, n (%)	2 (33%)	18 (31%)	NS
AH, n (%)	1 (17%)	30 (50%)	NS
Dyslipidemia, n (%)	1 (17%)	30 (50%)	NS
ACEi, n (%)	1 (17%)	15 (25%)	NS
Statins, n (%)	1 (17%)	28 (47%)	NS

Abbreviations: ACEi, angiotensin-converting enzyme inhibitors; AH, arterial hypertension; BMI, body mass index; BSA, body surface area; HF, heart failure; NS, non-significant.

**Table 2:** Clinical parameters of patients with DCM and ICM obtained prior to sampling of myocardium.

	DCM	ICM	*p*-Value
n	30	29	
Age (y), mean ± SEM	58 ± 6	57 ± 7	NS
Female gender, n (%)	3 (10%)	5 (17%)	NS
Smoking, n (%)	8 (26%)	10 (34%)	NS
BMI (kg/m^2^), mean ± SEM	26.7 ± 0.7	25.8 ± 0.6	NS
SBP (mmHg), mean ± SEM	108 ± 3	111 ± 2	NS
DBP (mmHg), mean ± SEM	73 ± 2	75 ± 2	NS
MAP (mmHg), mean ± SEM	85 ± 2	87 ± 9	NS
ICD/CRT, n (%)	18 (60%)	15 (52%)	NS
AH, n (%)	12 (40%)	18 (62%)	NS
Dyslipidemia, n (%)	8 (26%)	22 (75%)	0.0276
AF, n (%)	12 (40%)	14 (48%)	NS
CKD, n (%)	9 (30%)	8 (27%)	NS
Hyperuricemia, n (%)	8 (26%)	7 (24%)	NS
VT/VF, n (%)	18 (60%)	13 (45%)	NS
LVEF (%), mean ± SEM	22 ± 1	27 ± 2	NS
ACI, n (%)	7 (23%)	8 (27%)	NS
ARB, n (%)	13 (43%)	13 (44%)	NS
Neprilysin inhibitors, n (%)	12 (40%)	10 (34%)	NS
BB, n (%)	24 (80%)	22 (75%)	NS
Statins, n (%)	8 (26%)	20 (68%)	0.0017
Diuretics, n (%)	27 (90%)	26 (89%)	NS
MRA, n (%)	25 (83%)	24 (83%)	NS
Amiodarone, n (%)	18 (60%)	9 (31%)	0.0370
Anticoagulants, n (%)	21 (70%)	20 (68%)	NS
GFR (mL/min/1.73 m^2^), mean ± SEM	69 ± 4	72 ± 4	NS

Abbreviations: ACEi, angiotensin-converting enzyme inhibitors; AF, atrial fibrillation; AH, arterial hypertension; ARB, angiotensin II receptor blockers; BB, beta blockers; BMI, body mass index; BSA, body surface area; CKD, chronic kidney disease; CRT, chronic resynchronization therapy device; DBP, diastolic blood pressure; DCM, dilated cardiomyopathy; LVEF, left ventricular ejection fraction; GFR, glomerular filtration rate; ICD, intracardiac defibrillator; ICM, ischemic cardiomyopathy; MAP, mean arterial pressure; MRA, mineralocorticoid receptor antagonists; NS, non-significant; SBP, systolic blood pressure; SEM, standard error of the mean; VF, ventricular fibrillation; VT, ventricular tachycardia.

**Table 3:** Multilinear regression analyses results with HSPA9, OMA1, SPG7, and TOMM20 as a dependent variable. For each independent variable, both *t*- and *p*-values are presented to demonstrate the strength and statistical significance of their associations with protein concentrations. Statistically significant results (*p* < 0.05) are highlighted in red.

**Regression**	0.0009		0.2		0.001		0.03	
**Dependent variable**	HSPA9		OMA1		SPG7		TOMM20	
**Independent variable**	*t*	*p*	*t*	*p*	*t*	*p*	*t*	*p*
**4HNE**	3.76	0.0005	2.05	0.04	3.21	0.002	2.87	0.006
**Smoking**	1.80	0.08	0.58	0.57	1.64	0.11	0.85	0.40
**Sex**	1.46	0.15	0.2	0.84	2.38	0.02	0.59	0.56

**Table 4:** Age of patients undergoing heart transplantation or LVAD implantation (age_HTx/LVAD_) divided into groups with lower and higher protein expressions.

	UPRmt Protein	DCM	ICM	all
**age_HTx/LVAD_**	*p*-value	0.2246	0.2778	0.0635
	CLPP (Lo)	56.5 ± 1.8	55.7 ± 2.6	55.8 ± 1.6
	CLPP (Hi)	59.3 ± 1.3	58.9 ± 1.00	59.2 ± 0.8
**age_HTx/LVAD_**	*p*-value	0.4278	**0.0256**	0.2639
	HSP10 (Lo)	58.9 ± 1.8	54.6 ± 2.1	56.8 ± 1.4
	HSP10 (Hi)	57.1 ± 1.3	60.42 ± 1.0	59.0 ± 0.8
**age_HTx/LVAD_**	*p*-value	0.5000	0.0875	**0.0032**
	HSP60 (Lo)	57.2 ± 1.8	55.2 ± 1.9	55.3 ± 1.3
	HSP60 (Hi)	58.8 ± 1.3	59.7 ± 1.5	60.2 ± 0.9
**age_HTx/LVAD_**	*p*-value	0.7936	0.1487	0.3871
	mt-HSP70 (Lo)	58.3 ± 1.9	55.6 ± 1.7	57.0 ± 1.3
	mt-HSP70 (Hi)	57.7 ± 1.1	59.4 ± 1.9	58.5 ± 1.1
**age_HTx/LVAD_**	*p*-value	**0.0181**	0.1645	**0.0055**
	HTRA2 (Lo)	55.4 ± 1.9	55.6 ± 2.0	55.5 ± 1.3
	HTRA2 (Hi)	60.6 ± 0.8	59.3 ± 1.6	60.1 ± 0.9
**age_HTx/LVAD_**	*p*-value	0.2747	0.6744	0.7051
	LONP1 (Lo)	59.2 ± 1.8	56.9 ± 2.1	58.0 ± 1.4
	LONP1 (Hi)	56.8 ± 1.3	57.6 ± 1.5	57.0 ± 0.9
**age_HTx/LVAD_**	*p*-value	0.5940	**0.0094**	0.0673
	OMA1 (Lo)	57.5 ± 1.9	54.2 ± 2.0	56.2 ± 1.4
	OMA1 (Hi)	58.7 ± 1.3	60.8 ± 1.1	59.4 ± 0.8
**age_HTx/LVAD_**	*p*-value	0.0663	**0.0159**	**0.0027**
	54.2 ± 2.0	56.0 ± 1.8	54.4 ± 2.0	55.3 ± 1.4
	60.8 ± 1.1	60.1 ± 1.0	60.6 ± 1.7	60.2 ± 0.7
**age_HTx/LVAD_**	*p*-value	0.7458	0.0850	**0.0445**
	YME1L (Lo)	57.7 ± 1.8	54.9 ± 2.2	56.2 ± 1.4
	YME1L (Hi)	58.4 ± 1.3	59.7 ± 1.6	59.84 ± 0.9
**age_HTx/LVAD_**	*p*-value	0.2249	0.8490	0.3362
	TOMM20 (Lo)	59.4 ± 1.2	57.2 ± 1.8	58.8 ± 1.1
	TOMM20 (Hi)	56.6 ± 1.8	57.7 ± 2.0	57.0 ± 1.3

Bolded *p*-values are significant. Data are means ± SEM for each group. Half of the patients with a lower protein expression were assigned to the “Lo” group, and half of the patients with a higher lower protein expression were assigned to the “Hi” group. Data are presented for patients with dilated cardiomyopathy (DCM), ischemic cardiomyopathy (ICM), or combined (all).

## Data Availability

The data supporting the present findings are available from the corresponding author upon reasonable request. The data are not publicly available because authors did not requested that form the Institutional Review Board.
